#  SP1-induced lncRNA TINCR overexpression contributes to colorectal cancer progression by sponging miR-7-5p

**DOI:** 10.18632/aging.101839

**Published:** 2019-03-10

**Authors:** Shaojun Yu, Da Wang, Yingkuan Shao, Teng Zhang, Haiting Xie, Xiaomeng Jiang, Qun Deng, Yurong Jiao, Jinhua Yang, Cheng Cai, Lifeng Sun

**Affiliations:** 1 Surgical Oncology Department, The Second Affiliated Hospital, Zhejiang University School of Medicine, Hangzhou, Zhejiang 310009, China; 2 Cancer Institute, Key Laboratory of Cancer Prevention and Intervention, China National Ministry of Education, Key Laboratory of Molecular Biology in Medical Sciences, The Second Affiliated Hospital, Zhejiang University School of Medicine, Hangzhou, Zhejiang 310009, China; 3 Shanghai Tenth People's Hospital, and Department of Pharmacology, Tongji University School of Medicine, Shanghai 200092, China; 4 Digestive Department, Affiliated Hospital of Jiangsu University, Zhenjiang 212001, Jiangsu Province, China; 5 Department of Gastrointestinal Tumor Surgery, The Second Affiliated Hospital, Zhejiang University School of Medicine, Changxing Campus, People’s Hospital of Changxing County, Changxing, Zhejiang 313100, China; 6 Colorectal and Anal Surgery Department, Jinhua Hospital, Zhejiang Uiniversity School of Medicine, Jinhua, Zhejiang 321000, China

**Keywords:** SP1, TINCR, miR-7-5p, colorectal cancer

## Abstract

Mounting evidences have indicated that long noncoding RNAs (lncRNAs) play pivotal roles in human diseases, especially in cancers. Recently, TINCR was proposed to be involved in tumor progression. However, its role in colorectal cancer (CRC) remains elusive. In our study, we found that SP1-induced TINCR was significantly upregulated in CRC tissues and cell lines. Moreover, cox multivariate survival analysis revealed that high TINCR was an independent predictor of poor overall survival (OS). Functionally, knockdown of TINCR obviously suppressed CRC cells proliferation, migration and invasion in vitro, and inhibited CRC cells growth and metastasis in vivo. Mechanistically, we identified TINCR could act as a miR-7-5p sponge using RNA pull down, luciferase reporter and RIP assays. Furthermore, we showed that TINCR might promote CRC progression via miR-7-5p-mediated PI3K/Akt/mTOR signaling pathway. Lastly, we revealed that plasma TINCR expression was upregulated in CRC when compared to healthy controls and could be a promising diagnostic biomarker for CRC. Based on above results, our data indicated that TINCR might serve as a potential diagnostic and prognostic biomarker for CRC.

## Introduction

Colorectal cancer (CRC) is one of the most common malignancies in the world [1]. Despite improvement in medical technology over the past years, including surgical resection, chemotherapy, radiotherapy and molecular targeted therapy, the five-year survival rate of CRC still remains unsatisfied [1,2]. Therefore, exploring the molecular mechanisms underlying CRC initiation and development is urgently needed.

Long non-coding RNAs(lncRNAs) are a class of more than 200 necleotides in length with limited protein-coding potential [3]. Accumulating studies reported that many lncRNAs were dysregulated and involved in various biological process, such as cell proliferation, apoptosis, migration, invasion and metastasis [4,5].

TINCR, a lncRNA about 3.7kb, controls human epidermal differentiation and is frequently deleted and downregulated in human squamous cell carcinoma (SCC) [6,7]. Moreover, multiple evidences have shown that aberrant expression of TINCR is closely associated with various human cancers, for example, T-p Xu, et al. [8] reported that TINCR was overexpressed and regulated cell cycle progression and apoptosis via modulating CDKN1A/P21, KLF2 and CDKN2B/P15 expression in gastric cancer. Xiaochun Liu, et al. [9] reported that TINCR was downregulated and suppressed cell proliferation and invasion via regulating miR-544a/FBXW7 axis in lung cancer. These prompted us to investigate the role of TINCR in human CRC.

In this study, we found that TINCR was upregulated in CRC cells and tissue samples, and the upregulation of TINCR was induced by SP1. We also revealed that TINCR, acting as a miR-7-5p sponge, may promote CRC progression via miR-7-5p-mediated PI3K/ Akt/mTOR signaling pathway. Our findings uncover the role of TINCR as a regulator of CRC progression, and shed new light on our understanding of TINCR-mediated malignancy progression.

## RESULTS

### TINCR is obviously upregulated in CRC and increased TINCR expression predicts poor prognosis

**Figure 1:**
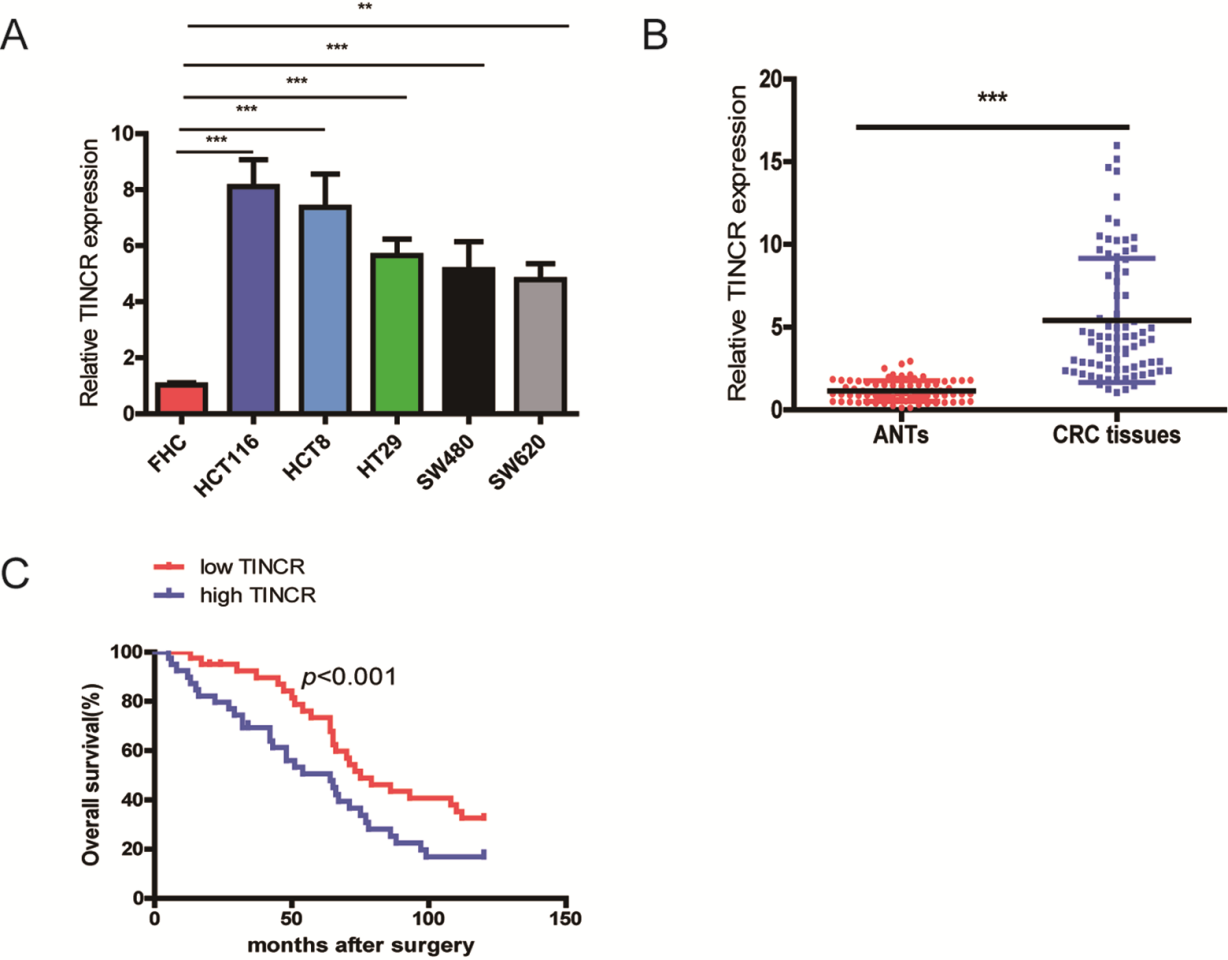
**TINCR is frequently upregulated in CRC tissues and cell lines and predicts poor prognosis.** (**A**) qRT-PCR analysis of TINCR expression in HCT116, HCT8, HT29, SW620, SW480 and FHC cells.GAPDH was used as an internal control. (**B**) qRT-PCR analysis of TINCR expression in 80 paired CRC tissues and and corresponding adjacent normal tissues. (**C**) Association of TINCR expression with OS (Kaplan-Meier plot). Data were shown as mean±S.D. of three independent experiments. **P<0.01, *** *P*<0.001.

Firstly, we investigated the expression level of TINCR in CRC cell lines and tissues. TINCR is significantly overexpressed in CRC cell lines (HCT116, HCT8, HT29, SW480 and SW620) in comparison with normal colon mucosal epithelial cell (FHC) (Fig. 1A). We also measured TINCR expression levels in CRC tissue samples and matched adjacent normal tissues (ANTs), and the results showed that TINCR expression was markedly higher in CRC tissue samples than that in the ANTs (Fig. 1B).

Next, we analyze the relationship between TINCR expression and clinicopathological characteristics of CRC patients, and total 80 CRC cases were divided into low-expressing group (n=40) and high-expressing group (n=40) based on median value of TINCR expression. As shown in Table 1, high-expressing of TINCR was obviously correlated with lymph node metastasis (*p*=0.012), differentiation (*p*=0.033) and TNM stage (*p*<0.001).

**Table 1:** Correlation between TINCR expression and different clinical characteristics.

**Patient characteristics**	**No. of Patients (%) (n=80)**	**TINCR expression**		***p* value**
		**High (%) (n=40)**	**Low (%) (n=40)**	
Age (years)				0.637
<60	27 (33.75%)	12 (30.0%)	15 (37.5%)	
≥60	53 (66.25%)	28 (70.0%)	25 (62.5%)	
Gender				0.367
Female	35 (43.75%)	15 (37.5%)	20 (50.0%)	
Male	45 (56.25%)	25 (62.5%)	20 (50.0%)	
Tumor location				0.073
Colon	41 (51.25%)	25 (62.5%)	16 (40.0%)	
Rectum	39 (48.75%)	15 (37.5%)	24 (60.0%)	
Tumor size (cm)				0.822
<5	36 (45.0%)	17 (42.5%)	19 (47.5%)	
≥5	44(55.0%)	23 (57.5%)	21 (52.5%)	
Differentiation				0.033
Well	15 (18.75%)	9 (22.5%)	6 (15.0%)	
Moderate	44 (55.0%)	13 (32.5%)	31 (77.5%)	
Poor	21 (26.25%)	18 (45.0%)	3(7.5%%)	
Serum CEA level (ng/mL)				0.261
<10	36 (45.0%)	21 (52.5%)	15 (37.5%)	
≥10	44 (55.0%)	19 (47.5%)	25 (62.5%)	
Local invasion				0.647
T1-T2	31 (38.75%)	17 (42.5%)	14 (35.0%)	
T3-T4	49 (61.25%)	23 (57.5%)	26 (65.0%)	
Lymph node metastasis				0.012
N0-N1	46 (57.5%)	17 (42.5%)	29 (72.5%)	
N2	34 (42.5%)	23 (57.5%)	11 (27.5%)	
TNM stage				<0.001
I-II	39(48.75%)	11(27.5%)	28(70.0%)	
III-IV	41(51.25%)	29(72.5%)	12(30.0%)	

Furthermore, CRC patients with high expression of TINCR predicted a poor overall survival (OS) using the analysis of Kaplan-Meier survival curve (*p*<0.001).

Univariate analysis revealed that differentiation, Lymph node metastasis, TNM stage and TINCR were predictors for poor OS. Multivariate analysis showed that upregulation of TINCR was obviously correlated with unfavorable OS (Table 2). These results suggest that TINCR upregulation as an early event in CRC development and have a pivotal role in CRC progression.

**Table 2:** Univariate and multivariate analysis for OS in patients with CRC.

**Characteristics**	**Multivariate analysis for OS**		**Univariate analysis for OS**	
	**HR (95% CI)**		**HR (95% CI)**	
Age (<60/≥60)	-	-	1.023(0.681-1.232)	0.332
Gender (Female/Male)	-	-	0.909(0.616-1.331)	0.215
Tumor location (colon/rectum)	-	-	0.918(0.661-1.421)	0.571
Tumor size (<5/≥5cm)	-	-	0.911(0.751-1.434)	0.314
Differentiation (well/moderate/poor)	1.878 (1.212-3.132)	0.019	3.125(2.019-5.111)	0.007
CEA (<10/≥10ng/mL)	-	-	0.799(0.516-1.114)	0.261
Location invasion (T1+T2/T3+T4)	-	-	1.091(0.781-1.298)	0.315
Lymph node metastasis (N0-N1/N2)	1.516(1.093-2.978)	0.012	1.902(1.311-3.391)	0.006
TNM stage (I-II/III-IV)	1.742 (1.065-3.145)	0.002	3.289 (2.141-4.995)	<0.001
TINCR (low/high)	0.459 (0.265-0.912)	0.027	0.414(0.226-0.624)	0.005

Abbreviations: CI, confidence interval; HR, hazard ratio.

### SP1 induces TINCR expression by functioning as a transcription factor

**Figure 2:**
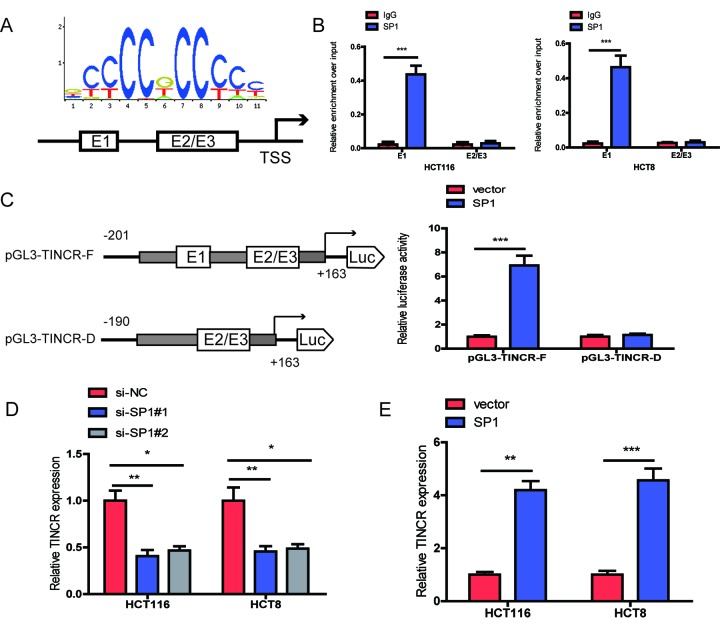
**SP1 is involved in TINCR upregulation.** (**A**)The predicted positions of putative SP1 binding motif in human TINCR promoter. (**B**) ChIP assays were employed to show direct binding of SP1 to endogenous TINCR promoter regions. (**C**) A luciferase reporter assay was performed by cotransfecting the full TINCR promoter(pGL3-TINCR-F) or deleted TINCR promoter fragment E1 (pGL3-TINCR-D) with SP1 or blank vector in 293T cells. (**D**, **E**) qRT-PCR analysis of TINCR expression levels following SP1 upregulation and knockdown. Data were shown as mean ±S.D. of three independent experiments. * *P*<0.05, **P<0.01, ***P<0.001.

The promoter region of TINCR was identified by JASPAR database (http://jaspardev.genereg.net/), and found that three putative SP1-binding sites at the sites E1(-163bp to-153bp, ACTCCGCCTCT), E2 (-88to -78bp, GCCCCGCCCCG) and E3 (-16 to -6bp, GGGGGCGGGCG) in the TINCR promoter (Fig. 2A). ChIP assay was performed to determine which region in the TINCR promoter mediated SP1-binding to the TINCR promoter. The ChIP data indicated that SP1 could bind to E1 sites (Fig. 2B). To further verify this result, we cloned the full promoter region of TINCR and E1 deleted promoter region into pGL3-basic reporter. Our results showed that the deletion of E1 sites significantly impaired the effect of SP1 on TINCR transcription activation (Fig. 2C), implying that SP1 could bind to the promoter of TINCR to regulate TINCR transcription.

Moreover, we upregulated SP1 by transfecting pcDNA3.1-TINCR and knocked down SP1 using an siRNA targeting SP1. Upon SP1 downregulation, TINCR expression was significantly decreased in HCT116 and HCT8 cells, while upon SP1 overexpression, TINCR expression was markedly increased in HCT116 and HCT8 cells (Fig. 2D, 2E).

### Silencing of TINCR inhibits CRC cells proliferation, migration and invasion in vitro

**Figure 3:**
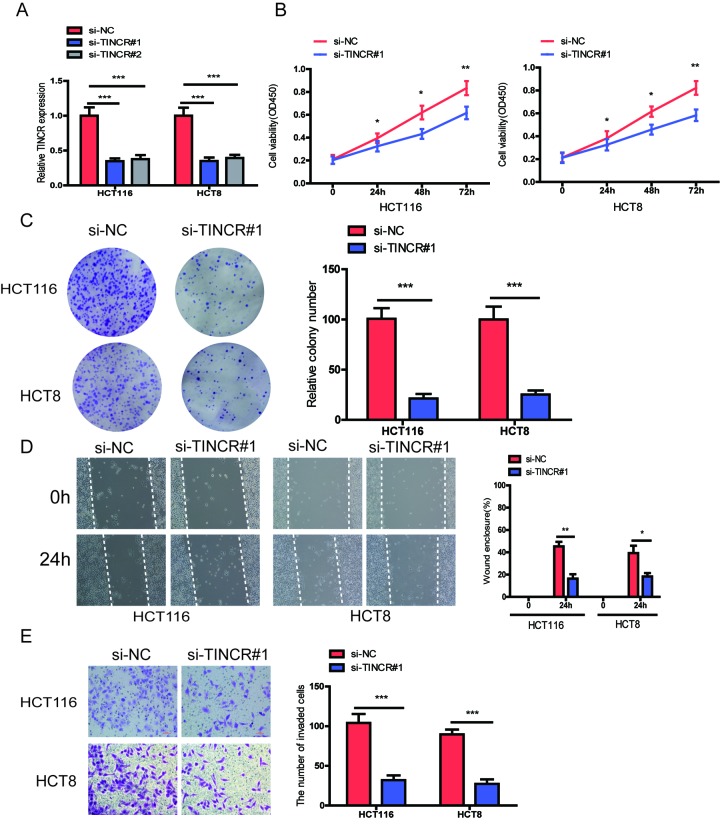
**TINCR knockdown suppressed CRC cells proliferation, migration and invasion.** (**A**) The relative expression of TINCR were detected after transfecting with siTINCR#1, siTINCR#2 or negative control (si-NC) in HCT116 and HCT8 cells. **(B**, **C**) Cells proliferation were evaluated in TINCR knockdown CRC cells using CCK-8 (**B**) and colony formation (**C**). (**D**, **E**) Wound healing (**D**) and transwell invasion (**E**) assays were performed to assess the ability of TINCR knockdown CRC cells. Data were shown as mean±S.D.. *P<0.05, **P<0.01. ***P<0.001.

To further explore the biological role of TINCR in CRC cells, we designed two siRNAs targeting TINCR to silence TINCR expression in HCT116 and HCT8 cells. The two siRNAs obviously decreased TINCR expression levels, and we chose si-TINCR#1 for the further study due to the higher inhibitory efficiency (Fig. 3A). CCK-8 assays showed that silencing of TINCR significantly inhibited CRC cell proliferation in HCT116 and HCT8 cells (Fig. 3B). The colony formation assay revealed that TINCR knockdown markedly suppressed colony forming ability of these two cells (Fig. 3C). Moreover, wound healing and transwell invasion assay demonstrated that TINCR knockdown obviously impeded HCT116 and HCT8 cells migration and invasion (Fig. 3D, 3E), respectively.

Based on above results, we preliminarily conclude that TINCR could retard the progression of CRC cells.

### Silencing of TINCR inhibits CRC cells growth and metastasis in vivo

**Figure 4:**
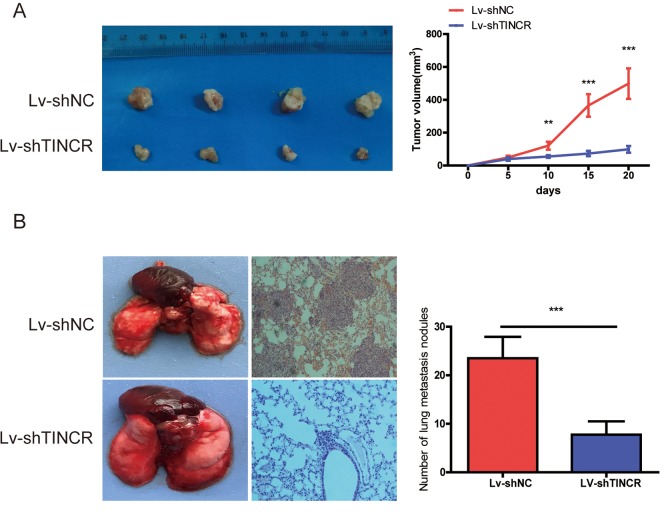
**TINCR knockdown inhibited tumor growth and metastasis.** (**A**) Subcutaneous implant model was established using TINCR stable knockdown HCT116 cells. The volume of xenograft tumors in two groups (n=4). (**B**) The number of metastatic nodules in the lungs of mice (three sections evaluated per lung) from two groups(n=4). Data are presented as the mean±S.D. **P<0.01. ***P<0.001.

To further explore the biological function of TINCR on CRC cells in vivo, we established the xenograft mice models by injecting TINCR stable knockdown HCT116 cells. As illustrated in Fig. 4A, TINCR depletion could inhibit tumor growth. We also established the lung metastasis model via tail vein injecting cells into nude mice (n=4 for each group), as shown in Fig 4B, an average of 24 lung metastatic nodules per mouse was detected in Lv-ShNC group, while only 8 were observed in Lv-shTINCR group. Overall, these results indicate that silencing of TINCR suppresses CRC cells growth and metastasis in vivo.

### TINCR can sponge miR-7-5p in CRC cells

**Figure 5:**
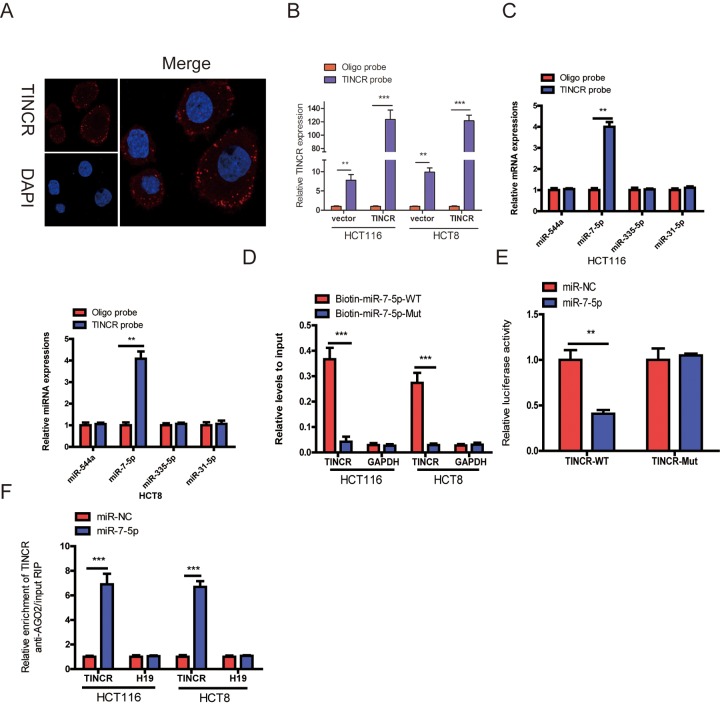
**TINCR functioned as a ceRNA by sponging miR-7-5p.** (**A**) RNA-FISH were employed to verify that TINCR was located mainly in the cytoplasm. (**B**) Lysates from HCT116 and HCT8 cells with TINCR overexpression were subject to biotinylated-TINCR pull-down assay and the expression of TINCR were measured by qRT-PCR. (**C**) The expression of four candidate miRNAs predicted by starbase database were quantified by qRT-PCR after the biotinylated-TINCR pull-down assay in HCT116 and HCT8 cells. (**D**) The biotinylated wild-type/mutant miR-7-5p was, respectively, transfected into HCT116 and HCT8 cells with TINCR overexpression. The expression of TINCR were measured by qRT-PCR after streptavidin capture. (**E**) Luciferase activity in HCT116 and HCT8 cells cotransfected with luciferase reporter containing TINCR sequences with wild-type or mutated miR-7-5p binding sites and miR-7-5p or its control. (**F**) Anti-AGO2 RIP was used in HCT116 and HCT8 cells overexpressing miR-7-5p, followed by qRT-PCR to assess the expression of TINCR or H19 (control) associated with AGO2. The data are presented as the mean±S.D. of three independent experiments. **P<0.01. ***P<0.001.

To detect the subcellular location of TINCR, the result of fluorescence in situ hybridization (FISH) indicated that TINCR mainly located in the cytoplasm (Fig. 5A).

Mounting evidences have proved that lncRNAs could function as competing endogenous RNA (ceRNA) to indirectly regulate miRNAs [11,12]. To explore whether TINCR has a similar mechanism in CRC cells, we selected four (miR-544a, miR-7-5p, miR-335-5p and miR-31-5p) candidate miRNAs through Starbase database (http://starbase.sysu.edu.cn/). A 3’terminal-biotinylated-TINCR probe was designed to investigate which miRNAs could interact with TINCR. The probe was proved to pull down TINCR in CRC cells and TINCR upregulation increased the pull-down efficiency (Fig. 5B), the results showed that miR-7-5p was the only miRNA that was abundantly pulled down by TINCR probe (Fig. 5C).

To further verify the interaction between them, TINCR- overexpressing cells were transfected with wild-type or mutant biotinylated miR-7-5p mimics, and the data suggested that wild-type miR-7-5p mimics captured more TINCR than mutant miR-7-5p mimics (Fig. 5D).

To further prove that TINCR could bind to miR-7-5p, the wild type sequence of TINCR (TINCR-WT) or its mutant sequence (TINCR-Mut) was cloned into the pmirGLO luciferase reporter, miR-7-5p significantly decreased luciferase activity of the pmirGLO-TINCR-WT vector, but failed to reduce luciferase activity of pmirGLO-TINCR-Mut vector. We preliminary revealed that TINCR was a target of miR-7-5p (Fig. 5E).

miRNAs bind their targets and exert the ability of translational repression or RNA degradation mainly in an AGO2-dependent manner [13,14]. To further determine whether TINCR binds to miR-7-5p in this manner, RIP was performed in CRC cells and confirmed the interaction between TINCR and miR-7-5p (Fig. 5F). These results strongly supported the idea that TINCR acted as a miR-7-5p sponge in CRC.

### miR-7-5p inhibition could reverse the inhibitory effect of TINCR downregulation

**Figure 6:**
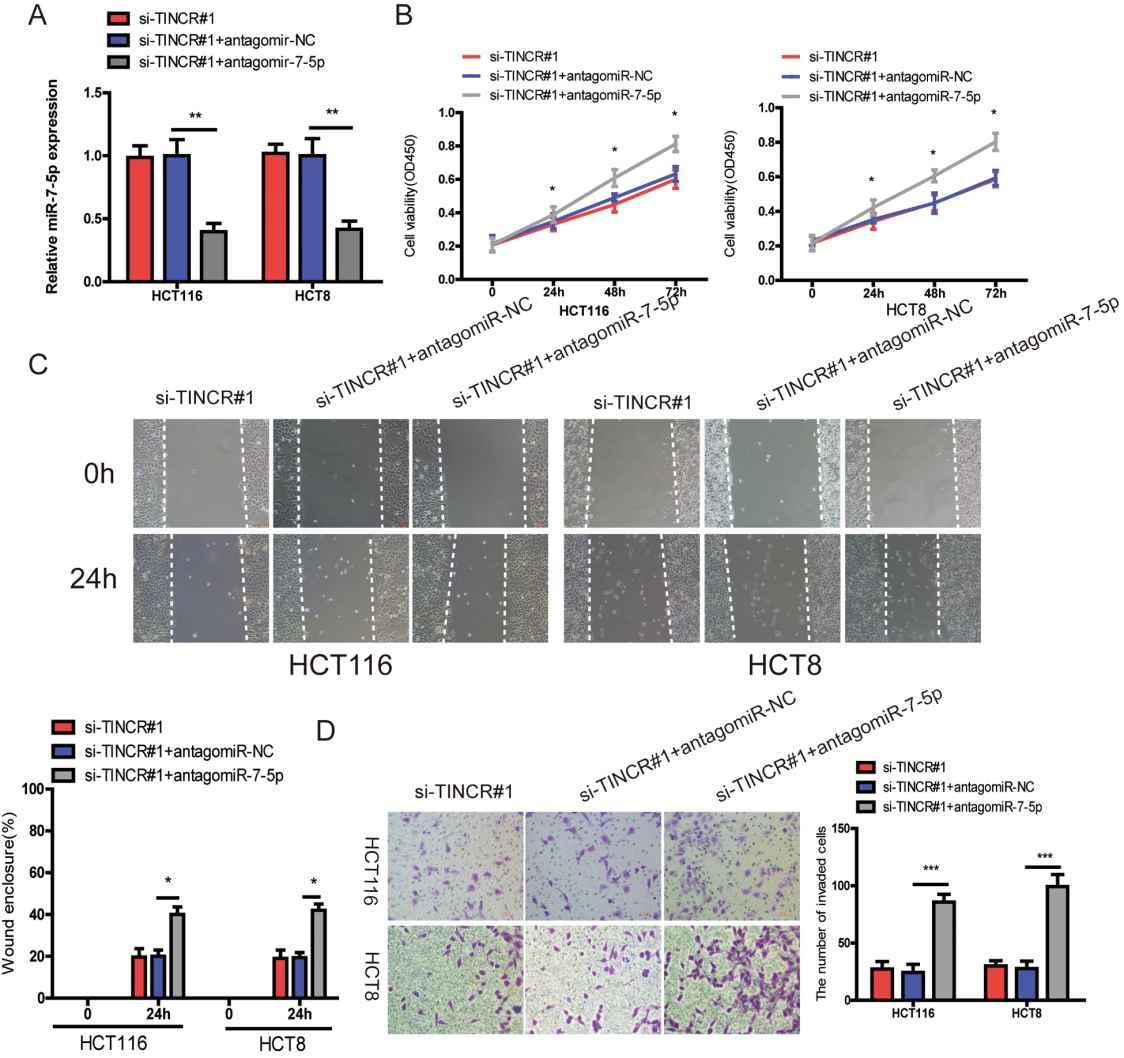
**miR-7-5p inhibition could reverse the inhibitory effect of TINCR downregulation.** (**A**) miR-7-5p was downregulated in TINCR knockdown CRC cells. (**B**, **C**, **D**) The cell proliferation, migration and invasion were assessed using CCK-8 (**B**), Wound-healing (**C**) and transwell invasion (**D**) assays in cells cotransfected with siTINCR#1 and antagomiR-7-5p or antagomiR-NC. The data are shown as the mean±S.D. of three independent experiments. *P<0.05, **P<0.01. ***P<0.001.

To further demonstrated that TINCR promoted CRC progression via sponging miR-7-5p. Firstly, we downregulated miR-7-5p expression in TINCR-silencing cells (Fig. 6A), and found that downregulation of miR-7-5p could partially counteract the decrease in proliferation (Fig. 6B), migration (Fig. 6C) and invasion (Fig. 6D) by the TINCR downregulation.

### TINCR may promote CRC progression via PI3K/Akt/mTOR signaling pathway.

**Figure 7:**
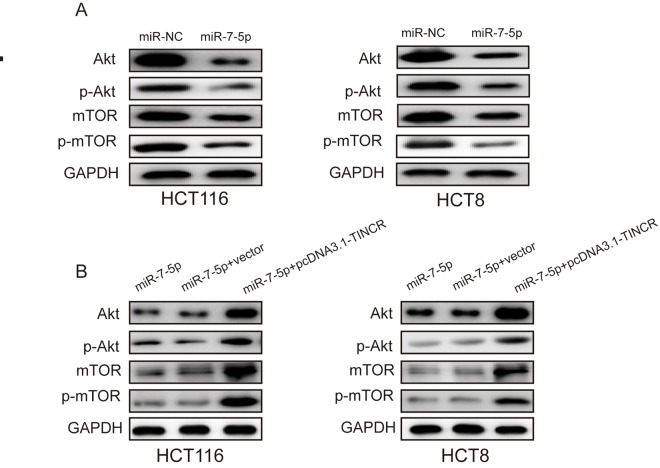
**TINCR may promote CRC progression via PI3K/Akt/mTOR pathway.** (**A**) miR-7-5p overexpression markedly inhibited the expression of Akt, p-Akt, mTOR and p-mTOR. (**B**) TINCR upregulation could restore the expression of Akt, p-Akt, mTOR and p-mTOR.

Previous studies have revealed that miR-7 regulated cell proliferation and metastasis via PI3K/Akt/mTOR signaling pathway [15–17]. Here, we found that miR-7-5p overexpression could suppress the expression of Akt and mTOR as well as phosphorylation of Akt and mTOR(p-Akt, p-mTOR) (Fig. 7A), while TINCR upregulation could restore the expression of Akt, mTOR, p-Akt and p-mTOR (Fig. 7B). Therefore, we could speculate that TINCR may promote CRC progression via miR-7-5p-mediated PI3K/Akt/mTOR signaling pathway.

### TINCR might be a potential diagnostic biomarker of CRC

**Figure 8:**
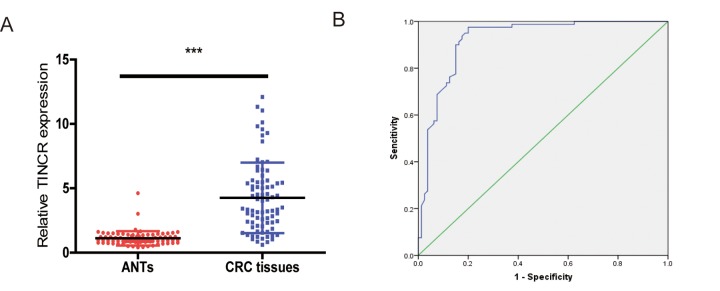
**Plasma levels of TINCR were upregulated in CRC patients.** (**A**) The relative levels of TINCR in CRC patients and healthy controls. All data were normalized to GAPDH. (**B**) ROC curves for TINCR in 80 patients with CRC and 80 healthy controls. ***P<0.001.

To determine whether plasma TINCR could be a potential biomarker for CRC, we compared plasma TINCR levels between healthy volunteers and CRC patients. The data showed that plasma TINCR level in CRC patients group was significantly higher than that in the healthy controls group (Fig. 8A). An ROC curve curve was performed for distinguishing healthy controls from patients with CRC. The results showed that the AUC was up to 0.922 (95% CI, 0.878-0.966; *p*<0.001). On the cutoff values from ROC curves, when the cutoff value of plasma TINCR was 1.815, sensitivity was 97.5%, and specificity was 80.0% (Fig. 8B).

## DISCUSSION

Increasing researches have shown that lncRNAs play pivotal roles in cancer initiation and progression, and dysregulated lncRNAs have also been identified in CRC [18,19]. Previous studies have reported that TINCR exerts discrepant function in various cancers. For example, Tongpeng Xu, et al. [20] reported that TINCR was aberrantly expressed in gastric cancer, and contributed gastric cancer progression through activating TINCR/STAU1/CDKN2B signaling axis. Zhijun Zhu, et al. [21] reported that TINCR was upregulated in non-small cell lung cancer and promoted NSCLC tumorigenesis and progression via BARF-activated MAPK pathway. Here, we showed that TINCR was upregulated in CRC tissues and cells, and TINCR overexpression was closely associated with differentiation, TNM stage, Lymph node metastasis and poor OS. Moreover, we found that silencing of TINCR significantly inhibited CRC cells proliferation, migration, invasion and metastasis.

SP1, as a transcription factor, could promote cancer progression through altering the expression of other genes, for example, Guanghua Liu, et al. [22] reported that SP1 could bind to the lncRNA-SNHG14 promoter region and promote its transcription. In this work, ChIP and luciferase reporter assays were performed and determine that SP1 could bind to TINCR promoter region, and then induce its transcription.

Increasing studies have verified that lncRNAs could regulate mRNA levels via competing for miRNAs, by acting as ceRNAs [23,24]. For example, Dong Liu, et al. [25] reported that lncRNA SPRY4-IT4 functioned as miR-101-3p sponge to promote bladder cancer cells growth and metastasis. Xiangsong Wu, et al. [26] reported that lncRNA-PAGBC competitively binds to miR-133b and miR-511 and promote tumor growth and metastasis in gallbladder cancer. In this study, we showed TINCR mainly located in the cytoplasm, bioinformatics analysis indicated that TINCR could interact with miR-7-5p, RNA pull down, RIP and luciferase reporter assay were performed to further verify that TINCR is able to bind to miR-7-5p. Furthermore, we found that downregulation of miR-7-5p could partially abrogate the decrease in proliferation, migration and invasion by the TINCR downregulation.

Previous studies have reported that Akt/mTOR signaling pathway played a critical role in various biological processes of cancers, such as proliferation, metastasis and survival [27–29]. Here, we revealed that TINCR may promote CRC progression via miR-7-5p-mediated PI3K/Akt/mTOR signaling pathway.

Recently, lncRNAs have been identified as novel biomarkers in various cancers [30,31]. Here, we tested the potential diagnostic value of plasma TINCR in differentiating people without CRC from CRC patients. We detected that plasma TINCR levels from patients with CRC were significantly higher than the levels from healthy controls. Our data suggested that plasma TINCR had a potential diagnostic value in CRC.

In summary, our study found that TINCR was upregulated in CRC tissues and cells, and was activated by transcription factor SP1. We also observed that TINCR, acting as a miR-7-5p sponge, promoted tumor growth, migration, invasion and metastasis in CRC. Our data will provide new insights into the underlying mechanism of CRC progression and suggested that TINCR might serve as a promising prognostic biomarker and a potential therapeutic target for CRC.

## MATERIALS AND METHODS

### Patients and tissue samples

A total of 80 CRC tissues and their matched adjacent normal tissues (ANTs) were obtained from CRC patients during operation in the Second Affiliated Hospital, Zhejiang University School of Medicine between January 2006 and July 2009. None patients achieve preoperative chemotherapy and radiotherapy before surgery. All these samples were placed immediately in liquid nitrogen and stored at -80°C until use. Informed consent was obtained from each patient and this study was approved by the Institutional Review Board of The Second Affiliated Hospital, Zhejiang University School of Medicine. Detailed patient information is listed in Table 1.

### Cell culture

Human normal colon epithelial cell (FHC) and CRC cell lines (HCT116, HCT8, HT29, SW620 and SW480) were obtained from ATCC. These cells were cultured in Dulbecco’s Modified Eagle Medium (DMEM, Gibco, USA) with 100U/ml penicillin, 0.1mg/ml streptomycin and 10% fetal bovine serum (FBS, Gibco, USA) at 37°C supplied with 5% CO_2_ atmosphere.

### Cell transfection and stable cell line construction

Si-TINCR (siTINCR#1, siTINCR#2), si-SP1(si-SP1#1, siSP1#2), miR-7-5p agomir, miR-7-5p antagomir and their corresponding negative control (NC) were obtained from Genepharma (Shanghai, China) and the sequences of siRNAs were listed in Table S1. To overexpress TINCR, human TINCR was synthesized and cloned into pcDNA3.1 vector. Transfections were performed using Lipofectamine 2000(Invitrogen) in accordance with the manufacture’s protocol.

To prove the function of TINCR in vivo, we constructed stable TINCR knockdown cells using lentivirus-mediated short hairpin RNA (Lv-shTINCR or LV-shNC) against the more effective siRNA target site (si-TINCR#1) or its negative control (si-NC). HCT116 cells were infected with concentrated virus, then the cells were selected by treatment of puromycin (2µg/ml).

### Luciferase reporter assay

For the promoter of TINCR luciferase reporter assay, the wild-type (WT) and mutant (Mut) promoters of TINCR were synthesized and cloned into PGL3-basic vectors (GeneCreat, China), and cotransfected with SP1 plasmid into 293T cells.

For TINCR and miR-7-5p luciferase reporter assay, the TINCR sequences containing WT or Mut miR-7-5p binding sites (BS) were respectively synthesized and cloned into pmirGLO luciferase vector (GeneCreat, China), then cotransfected with miR-7-5p mimics or its negative control(miR-NC) into 293T cells. Cells were harvested at 48 h after transfection, and the luciferase activity was detected using the dual-luciferase reporter assay system (Promega).

### Fluorescent in situ hybridization (FISH)

FISH assay was performed using a Ribo™ lncRNA FISH Probe Mix and Ribo™ Fluorescent In Situ Hybridization Kit and (Ribo, China) following the manufacturer’s protocols.

### Chromatin immunoprecipitation (ChIP) assays

The ChIP assay kit (Beyotime, China) was used to conduct ChIP assays. In brief, the cells were fixed with 1% formaldehyde solution for 20 min and quenched with 0.125M glycine for 10 min. DNA fragments ranging from 200 bp to 300 bp were generated using sonication. Antibodies including anti-SP1(ab227383, Abcam) and IgG were employed for each immunoprecipitation. The precipitated DNA was analyzed using qPCR. The primer sequences were listed in Table S2. 

### RNA immunoprecipitation

An EZMagna RNA immunoprecipitation (RIP) Kit (Millipore, Billerica, USA) was used according to the manufacturer’s protocol. Antibody (anti-AGO2) for RIP assay was from Abcam.

### Biotinylated RNA pull-down assay

The pull-down assay with biotinylated RNA was performed as described previously [10]. In brief, for TINCR pulled down miRNAs, the biotinylated-TINCR probe was incubated with C-1 magnetic beads (Life Technology) to generate probe-coated beads, incubated with sonicated cells at 4 °C overnight,followed by eluted and qRT-PCR. For miR-7-5p pulled down TINCR, cells with TINCR overexpression were transfected with biotinylated miR-7-5p mimics or mutant using Lipofectamine 2000. Then the cells were harvested, lysed, sonicated, and incubated with C-1 magnetic beads (Life Technologies), followed by washed and qRT-PCR.

### RNA isolation and qRT-PCR

Total RNA from tissue samples, cells and plasma were isolated using TRIzol reagent (Invitrogen). The miR-7-5p levels were quantified using Hairpin-itTM MicroRNAs Quantitation PCR (GenePharma, China), TINCR levels were measured by SYBR Green PCR kit (Takara, Japan). U6 was selected as reference gene for miR-7-5p and GAPDH was chosen as an internal control for TINCR. The relative expression of TINCR or miR-7-5p using the 2^-ΔΔCt^ method. The primer sequences were shown in Table S3.

### Western blot

Proteins were extracted from cells using RIPA lysis buffer. Equal amounts of protein (30µg) were separated by SDS-PAGE and transferred onto polyvinylidene fluoride membranes (PVDF), and incubated with primary antibody at 4 °C overnight. Afterwards, the membranes were incubated with secondary antibody for 1 h. The blots were detected by using enhanced chemiluminescence reagents (ECL, KeyGEN BioTECH, China).

### Cell counting kit-8 assay

CCK-8 assay was employed to evaluate the proliferative ability of CRC cells following manufacturer’s protocol. In brief, 1×10^3^ transfected cells were seeded into 96-well plates and treated with 10 µl CCK-8 solution. Absorbance was detected at 450 nm by using a microplate reader (Bio-Tek Instruments Inc., Winooski, VT, USA).

### Colony formation assay

1000 transfected cells were plated into 6-well plates and cultured for two weeks. The colonies were fixed with 100% methanol for 15 min and stained using 0.1% crystal violet for 20 min at room temperature.

### Wound-healing assay

Wound-healing assay was used to assess cell migration. In brief, transfected cells were plated into six well plates (3×10^5^ cells/well) and grown to about 90% conﬂuence. Cells were scratched by using 200 µL pipette tips, and then washed with PBS. Cells were further cultured with a medium containing 1% FBS for 24 h. Images were detected using an inverted microscope (Nikon Corporation, Tokyo, Japan) at 100×magnifcation.

### Transwell invasion assay

The cell invasion was performed using a 24-well transwell chamber (Corning, NY, USA. Cells were plated into upper chamber precoated with 2% Matrigel (BD Biosciences, USA). The lower chambers were loaded with 500µl DMEM containing 20% FBS. The invaded cells were fixed with 100% methanol for 15 min and stained using 0.1% crystal violet for 20 min at room temperature. The number of invaded cells were counted by a microscope (Nikon Corporation, Tokyo, Japan) at 200×magnification.

### In vivo experiments

All animal experiments were approved by the animal care Committee of The Second Affiliated Hospital, Zhejiang University School of Medicine.

For xenograft tumor model, 1×10^7^ TINCR stable knockdown HCT116 cells were subcutaneously into the the armpit region of 8 six-week old male BABL/c nude mice which were randomly divided into two groups (n=4 each group) into the tumors. The tumors were measured every 5 days. The volume of tumors was calculated using the following equation: V=(L×W^2^)/2, L is the length and W is the width of tumor.

For metastasis assay, 2 × 10^6^ stable knockdown HCT116 cells were injected into 8 six-week-old male BALB/c nude mice (n=4 for each group) which were randomly divided into two groups (n = 4 for each group). Six weeks later, the mice were sacrificed, and their lungs were removed and stained by Hematoxylin and Eosin (HE) Staining.

### Statistical analysis

All experiments were performed in triplicate. The data are presented as mean ± S.D., and statistical analysis were conducted using SPSS version 17.0. One-way analysis of variance (ANOVA) and the Student’s t-test were used to estimate the differences between groups. χ2 test or Pearson’s Mann-Whitney U test was employed to analyze the relationship between TINCR expression and clinicopathological factors. Kaplan-Meier method was performed to evaluate overall survival (OS). The survival curves were compared with log-rank test. Cox proportional hazards model was applied to perform multivariate analysis and calculate the 95% confidence interval (95% CI). *p*<0.05 was considered to be statistically significant.

## Supplementary Material

Supplementary Tables
