# Supplementary material for: SP1-induced lncRNA TINCR overexpression contributes to colorectal cancer progression by sponging miR-7-5p
Source: Aging (Albany NY). 2019 Mar 10;11(5):1389–403. doi: 10.18632/aging.101839 (PMC6428101; doi:10.18632/aging.101839)
Supplement: Supplementary Tables [file aging-11-101839-s001.pdf]

SUPPLEMENTARY TABLES

Table S1. Primers used for qRT-PCR.

TINCR-F	TGTGGCCCAAACTCAGGGATACAT
TINCR-R	AGATGACAGTGGCTGGAGTTGTCA
GAPDH-F	GCTCTCTGCTCCTCCTGTTC
GAPDH-R	ACGACCAAATCCGTTGACTC

Table S2. QChIP analysis of the TINCR promoter for SP1 occupancy.

E1 region	F	TGACCTCGCTGATGGCTCT
	R	TCAGGCGTCCGCTCCCCACT
E2 /E3region	F	TGAGGGGACCGTGGCA
	R	TGGTAGCGCTTCCAGCGCGACA

Table S3. siRNAs oligonucleotides.

si-NC	UUCUCCGAACGUGUCACGUdTdT
si-SP1#1	CAGCGUUUCUGCAGCUACCUUGACU
si-SP1#2	GACAGGUCAGUUGGCAGACUCUACA
si-TINCR#1	UAUUCCUUCAGCCAGUACCCAGGUC
si-TINCR#2	UUUCCAAGGUGGCACAGUGCUUUCC
